# Supplementary material for: Early-Life Colonization by Anelloviruses in Infants
Source: Viruses. 2022 Apr 22;14(5):865. doi: 10.3390/v14050865 (PMC9146212; doi:10.3390/v14050865)
Supplement: Supplementary file 1 [file viruses-14-00865-s001.zip › viruses-1672890 - Supplementary Figures_23-03.pdf]

Supplementary Figures

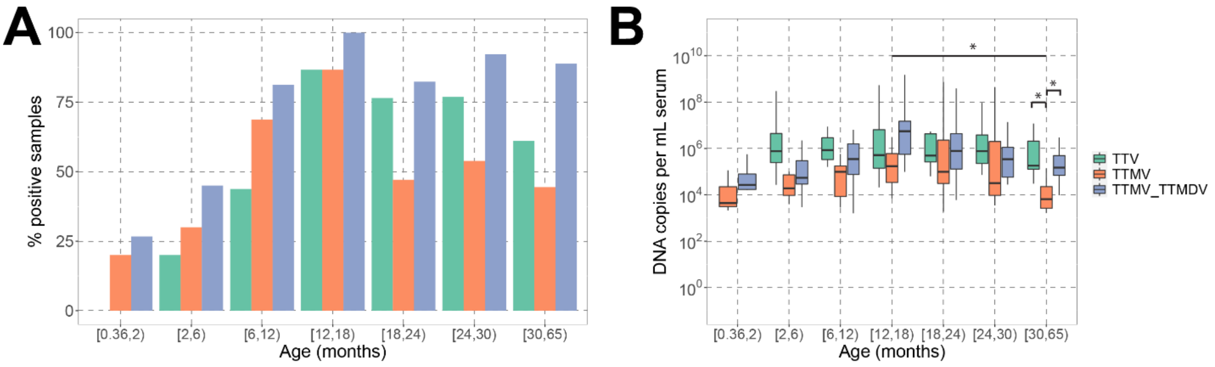

Figure S1: Anellovirus prevalence and concentration at different children ages. (**A**) Percentage of samples positive in all three qPCRs in 7 age groups. (**B**) Concentrations were detected by the qPCRs in the age groups. The significant differences in AV DNA concentrations between the age groups are marked with asterisks. The significance was measured by Wilcoxon rank-sum test; explanations of the symbols: * P ≤ 0.05, ** P ≤ 0.01, *** P ≤ 0.001, **** P ≤ 0.0001.

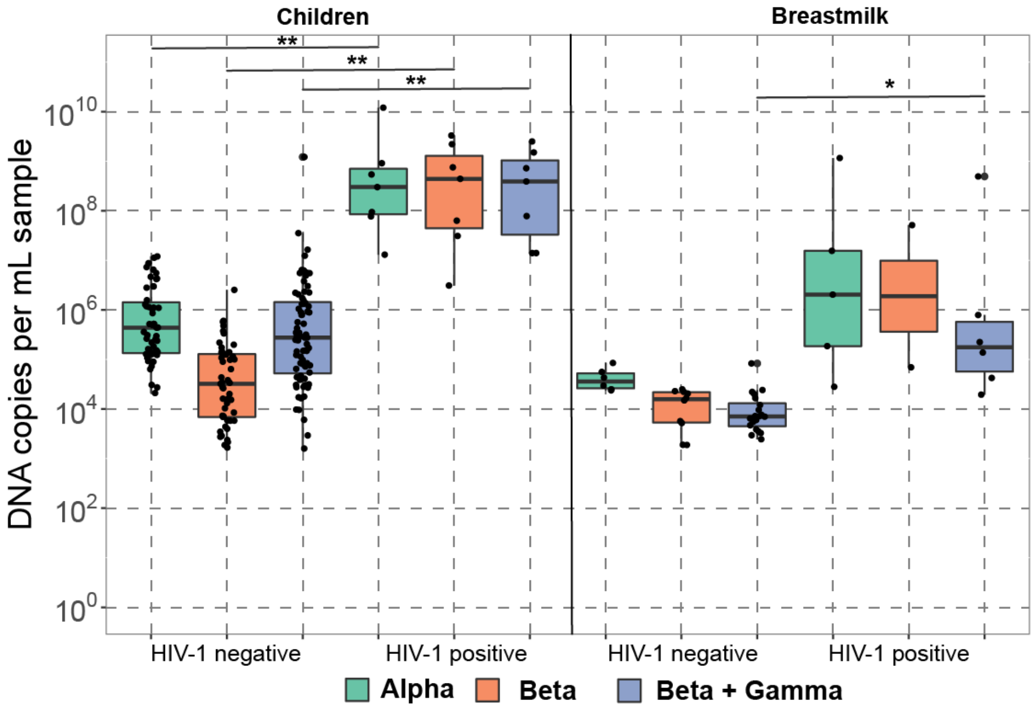

Figure S2: Comparison of AV DNA concentration in samples from HIV-1 positive subjects with HIV-1 negative subjects. The significant differences in AV DNA concentrations between the age groups are marked with asterisks. The significance was measured by Wilcoxon rank-sum test; explanations of the symbols: * P ≤ 0.05, ** P ≤ 0.01, *** P ≤ 0.001, **** P ≤ 0.0001.
